# Adaptive discrimination between non-tuberculous mycobacteria by *Caenorhabditis elegans*

**DOI:** 10.17912/micropub.biology.002170

**Published:** 2026-07-08

**Authors:** Rashi Mittal, Abinaya P., Rajesh S Gokhale, Arnab Mukhopadhyay

**Affiliations:** 1 National Institute of Immunology, Aruna Asaf Ali Marg, New Delhi 110067, India; 2 Molecular Aging Laboratory, National Institute of Immunology, Aruna Asaf Ali Marg, New Delhi 110067, India; 3 Indian Institute of Science Education and Research Pune, Pune, MH, IN

## Abstract

*Caenorhabditis elegans* modifies its behaviour in response to bacterial cues, but its ability to distinguish between closely related environmental bacteria remains unclear. Here, we examine responses to non-tuberculous mycobacteria, the opportunistic pathogen *Mycobacterium abscessus* and the non-pathogenic *Mycobacterium smegmatis .* While *Escherichia coli* OP50 was the preferred choice when available, worms showed distinct, time-dependent responses to the mycobacterial species. Notably, prior exposure to *M. smegmatis* drove faster and stronger avoidance of *M. abscessus* than exposure to the pathogen itself. These findings thus show that *C. elegans* can rapidly and adaptively discriminate between closely related mycobacteria, highlighting how prior experience shapes behavioural decisions.

**Figure 1. Prior conditioning differentially shapes bacterial preference in C. elegans:** **(A)** Schematic illustration of the binary food choice assay used to assess bacterial preference behaviour in *C. elegans* **(B-D)** Wild-type (WT) worms conditioned on OP (B), MA (C), or MS (D) exhibited a strong preference for OP when it was presented as one of the choices. **(E–G)** Differences in bacterial choice kinetics emerged when OP-, MA-, or MS-conditioned worms were presented with a choice between MA and MS. Statistical significance was determined using the Kruskal–Wallis test followed by Dunn's multiple comparisons test. Each dot represents an independent experimental replicate. Experiments were performed in triplicate, with 10–15 technical replicates per conditioning group. Abbreviations: OP, *Escherichia coli* OP50; MA, *Mycobacterium abscessus* ; MS, *Mycobacterium smegmatis* .

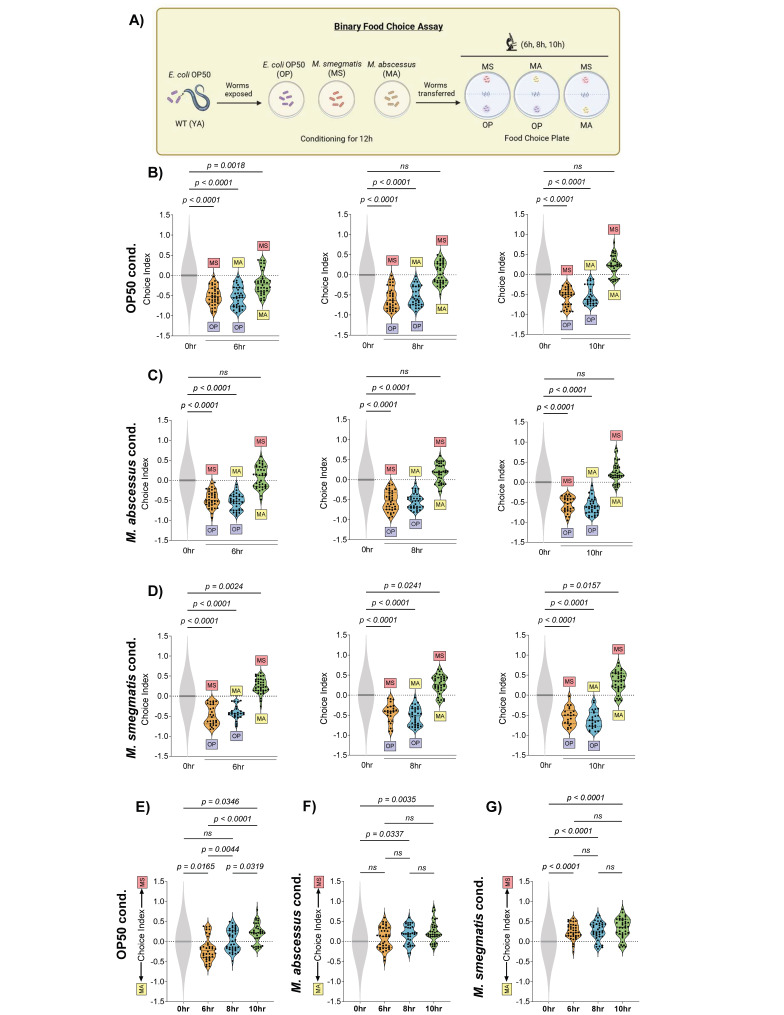



## Description

Animals that inhabit microbe-rich environments must continuously evaluate available microbial resources to balance nutritional benefits with potential pathogenic risks. This challenge is particularly relevant for *Caenorhabditis elegans* , a soil-dwelling nematode that thrives in microbially dense, decomposing environments, where it encounters diverse and fluctuating bacterial communities that influence its behaviour and physiology ( **Samuel et al., 2016; Berg et al., 2016; Schulenburg & Félix, 2017** ). Previous studies have established that *C. elegans* can discriminate between bacterial species and preferentially accumulate on higher-quality diets using a combination of sensory cues and post-ingestive feedback ( **Shtonda and Avery 2006** ). In addition, worms can modify their behavioural responses following exposure to pathogenic bacteria through experience-dependent avoidance mechanisms ( **Zhang et al. 2005; Pradel et al. 2007; Meisel and Kim 2014** ).

Behavioural responses of *C. elegans* to pathogenic bacteria have been extensively characterized in systems such as *Pseudomonas aeruginosa* , *Serratia marcescens* , and *Enterococcus faecalis* , where worms exhibit both innate and learned avoidance behaviours ( **Zhang et al. 2005; Pradel et al. 2007; Meisel and Kim 2014; Garsin et al. 2001** ). However, behavioural interactions between *C. elegans* and environmental mycobacteria remain unexplored. Non-tuberculous mycobacteria (NTMs) are ubiquitous environmental microorganisms that include both opportunistic pathogens and non-pathogenic species ( **Falkinham 2009** ). Among these, *Mycobacterium abscessus* (MA) is a rapidly growing opportunistic pathogen associated with pulmonary and soft-tissue infections ( **Lopeman et al. 2019; Bryant et al. 2016** ), whereas *Mycobacterium smegmatis* (MS) is generally considered non-pathogenic and is widely used as a model organism for mycobacterial biology ( **Snapper et al. 1990** ). Whether *C. elegans* can distinguish between such closely related mycobacterial species remains unknown.

To address this, we examined behavioural responses of *C. elegans* using a binary choice assay ( **Fig. 1A** ). Worms were raised on *Escherichia coli* OP50 (OP) from the L1 to young adult stage, washed, and subsequently conditioned on OP, MA, or MS for 12 hours. Animals were then transferred to the centre of assay plates (0 hours), and their distribution between bacterial lawns was monitored over time (6, 8, and 10 hours).

Across all experimental conditions, when OP was presented as one of the available bacterial options, worms consistently accumulated on OP50 lawns, irrespective of prior conditioning ( **Fig1. B, C, D** ). These observations indicate that both mycobacterial species are perceived as less favourable relative to the standard laboratory diet.

In contrast, when worms were restricted to mycobacterial choices, a striking and condition-dependent behavioural pattern emerged. Worms conditioned on OP initially displayed a significant preference for MA at the early time point of 6 hours ( **Fig1. E** ). However, this preference was transient; over time, worms progressively redistributed toward MS, with a clear shift observed by 10 hours ( **Fig1. E** ). These dynamics indicate that worms conditioned on OP initially favour MA, but following sampling and likely post-ingestive evaluation, subsequently avoid MA and accumulate on MS, the non-pathogenic mycobacteria.

Prior conditioning on mycobacterial species, however, markedly altered both the timing and direction of this behavioural transition. Worms conditioned on MA did not exhibit immediate avoidance of the pathogenic bacterium. Instead, they initially distributed between MA and MS, consistent with continued sampling of the bacterial choice given. And then a gradual redistribution toward MS was observed, becoming significant by 8 hours and strengthening further by 10 hours ( **Fig1. F)** . Thus, prior exposure to MA does not eliminate early sampling behaviour, but it enables faster discrimination compared to OP-conditioned animals.

In contrast, worms conditioned on MS exhibited a fundamentally different response. These animals showed an immediate and sustained accumulation on MS, with minimal occupancy of MA from the earliest time points measured. Notably, this shift was evident as early as 6 hours ( **Fig1. G),** indicating a rapid and robust behavioural bias against the pathogenic species.

This differential response across conditioning states reveals an unexpected hierarchy in behavioural adaptation. While conditioning on the pathogenic bacterium results in a gradual shift away from it, conditioning on the non-pathogenic bacterium drives a more immediate and pronounced avoidance of the pathogenic species. In other words, prior exposure to a “favourable” microbial environment accelerates discrimination more effectively than exposure to a pathogenic one.

Taken together, our findings demonstrate that *C. elegans* can discriminate not only between standard laboratory bacteria and alternative species, but also between closely related mycobacterial species that differ in pathogenic potential.

To our knowledge, this represents the first demonstration of adaptive behavioural discrimination between non-tuberculous mycobacterial species by *C. elegans* . This discrimination is rapid, strongly shaped by prior experience, and unexpectedly more efficient following exposure to a non-pathogenic mycobacterium than a pathogenic one. These findings thus establish a simple and tractable framework for investigating how animals evaluate closely related environmental microbes and dynamically adjust their behaviour to optimize survival in complex microbial environments.

## Methods

**Strains and worm maintenance**

Wild-type *Caenorhabditis elegans* ( N2 Bristol) were maintained under standard conditions at 20°C on nematode growth medium (NGM) plates seeded with *Escherichia coli* OP50 (OP). Synchronized L1 larvae were obtained by standard bleaching methods and cultured on OP50 until the young adult (YA) stage. For conditioning experiments, YA worms were transferred to NGM plates seeded with OP, *Mycobacterium smegmatis* (MS), or *Mycobacterium abscessus* (MA) and maintained on the respective bacterial lawns for 12 hours, then subjected to binary food choice assays.

**Bacterial culture conditions**

OP was grown on Luria-Bertani (LB) agar plates. Single colonies were inoculated into LB broth and grown overnight at 37°C with shaking (180 rpm). Secondary cultures were initiated with 2% primary inoculum and grown to mid-log phase (OD₆₀₀ = 0.6-0.8). MA and MS were maintained on Middlebrook 7H11 agar supplemented with 0.5% glycerol and 10% oleate-albumin-dextrose-catalase (OADC). Primary cultures were inoculated into Middlebrook 7H9 broth supplemented with 0.2% glycerol, 10% OADC, and 0.25% Tween-80 and grown at 37°C with shaking (180 rpm). Secondary cultures were grown to mid-log phase (OD₆₀₀ = 0.4-0.6). Bacterial cultures were harvested by centrifugation and resuspended in 1×M9 buffer to one-tenth of the original culture volume. For plate preparation, ~500 µl of OP and ~300 µl of MA and MS bacterial suspension were spread onto NGM plates and allowed to dry at room temperature for ~24 hours.

**Binary Food Choice Assay**

Food-choice assay plates were prepared by growing *E. coli* OP50 secondary cultures to an OD₆₀₀ of 0.6–0.8, and MA and MS cultures to an OD₆₀₀ of 0.4–0.6. Bacterial cultures were harvested by centrifugation and resuspended in 1× M9 buffer to one-tenth of the original culture volume. For assay plate preparation ( OP50 vs. MS, OP50 vs. MA, and MA vs. MS), 50 µl of each bacterial suspension was spotted at opposite ends of 90 mm agar plates. Plates were incubated at 20°C for ~16-24 hours to allow the establishment of bacterial lawns. Synchronized young adult (YA) worms, initially grown on OP50 , and conditioned on OP50 , MA, or MS for 12 hours, were collected and washed three times with 1× M9 buffer to remove residual bacteria. Approximately 80–100 worms were then transferred to the centre of each assay plate.Plates were incubated at 20°C, and worm distribution was scored at 6, 8, and 10 hours based on their location on the respective bacterial lawns.

**Data analysis**

Behavioural preference was quantified using a preference index (I), calculated as:

$$I=\frac{N_{T}-N_{c}}{N_{\text{total}}}$$

where *N _T_* represents the number of worms on the test bacterial lawn, *N _c_* represents the number of worms on the alternative lawn, and *N _total_* is the total number of worms scored.

**Statistical Analysis**

Statistical analysis was done using GraphPad Prism 10. P-values less than 0.05 were taken as statistically significant.

## Reagents

**Table:** 

**Strain Used**	**Genotype**	**Source**	**Description**
N2	Wild-type	CGC	Standard laboratory strain
*Escherichia coli*	OP50	CGC	Standard laboratory strain
*Mycobacterium smegmatis*	Wild-type	ATCC700084	Standard laboratory strain
*Mycobacterium abscessus*	Wild-type	ATCC19977	Standard laboratory strain

## Data Availability

Description: Source data file. Resource Type: Dataset. DOI: https://doi.org/10.22002/egn6r-9ss43
